# Supplementary figures and images for: Do auditory deviants evoke cortical state changes under anaesthesia? A proof‐of‐concept study
Source: Exp Physiol. 2025 Nov 25;111(4):2148–62. doi: 10.1113/EP093378 (PMC13140387; doi:10.1113/EP093378)

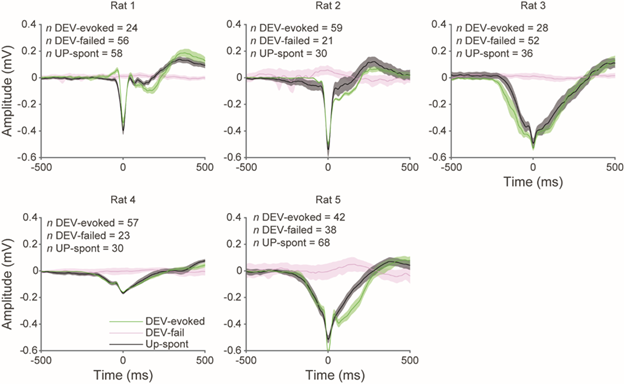

Supplement: Supplementary file 2 — Figure S1. DEV‐evoked and spontaneous Up stateshave similar dynamicsMean ± 95% CI plots of Deviant‐evoked Up states (green) and spontaneous Up states (black) show similar responseprofiles. In comparison, when deviants failed (pink) to evoke an Up state, noother response is observed. Data from mPFC left electrodes, temporally alignedrelative to negative peaks to allow shape comparison. [file EPH-111-2148-s003.tif]

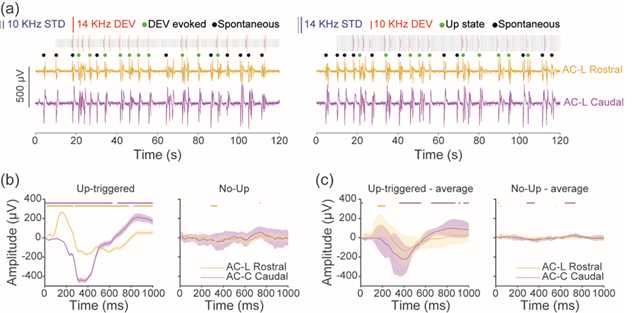

Supplement: Supplementary file 3 — Figure S2. Comparison of rostral and caudal ACrecordings. A) LFPtraces from AC‐L Rostral (yellow) and AC‐L Caudal (magenta) electrodes for anexample animal. For each animal, a classical oddball paradigm was presentedwith a 500 ms SOA and 10% DEV probability. Stimuli length was 75 ms, 10% DEVprobability, 82 minutes. Up state initiations were identified and classified aseither evoked (AC‐L Rostral response occurring 100‐500ms after a DEV; greendots) or spontaneous (black dots). B) Mean ± 95% CI plots of DEVresponse from an example animal at different electrodes sites when the DEV isfollowed by an Up state (left) or not followed by an Up state (right). Yellowand magenta bars indicate the presence of significance from 0 for AC‐L Rostraland AC‐L caudal, respectively (one sample t‐test, t(42) p<0.05). [file EPH-111-2148-s002.tif]
